# Localisation and regulation of cholesterol transporters in the human hair follicle: mapping changes across the hair cycle

**DOI:** 10.1007/s00418-020-01957-8

**Published:** 2021-01-06

**Authors:** Megan A. Palmer, Eleanor Smart, Iain S. Haslam

**Affiliations:** 1 Department of Biological Sciences, School of Applied Sciences, University of Huddersfield, Huddersfield, HD1 3DH UK; 2 Centre for Dermatology Research, School of Biological Sciences, University of Manchester and NIHR Biomedical Research Centre, Manchester, M13 9PT UK

**Keywords:** Hair follicle, Cholesterol, ABC transporters, Hair cycle, Keratinocytes, Liver X receptors

## Abstract

Cholesterol has long been suspected of influencing hair biology, with dysregulated homeostasis implicated in several disorders of hair growth and cycling. Cholesterol transport proteins play a vital role in the control of cellular cholesterol levels and compartmentalisation. This research aimed to determine the cellular localisation, transport capability and regulatory control of cholesterol transport proteins across the hair cycle. Immunofluorescence microscopy in human hair follicle sections revealed differential expression of ATP-binding cassette (ABC) transporters across the hair cycle. Cholesterol transporter expression (ABCA1, ABCG1, ABCA5 and SCARB1) reduced as hair follicles transitioned from growth to regression. Staining for free cholesterol (filipin) revealed prominent cholesterol striations within the basement membrane of the hair bulb. Liver X receptor agonism demonstrated active regulation of ABCA1 and ABCG1, but not ABCA5 or SCARB1 in human hair follicles and primary keratinocytes. These results demonstrate the capacity of human hair follicles for cholesterol transport and trafficking. Future studies examining the role of cholesterol transport across the hair cycle may shed light on the role of lipid homeostasis in human hair disorders.

**Supplementary Information:**

The online version contains supplementary material available at 10.1007/s00418-020-01957-8.

## Introduction

Cholesterol plays a vital role in cutaneous physiology, being integral to epidermal barrier function (Feingold 2009; Wertz 2000) and acting as a metabolic precursor in steroid hormone synthesis (Payne and Hales 2004; Slominski et al. 2013; Thiboutot et al. 2003). Cholesterol modifications are also vital for signal transduction in the Wnt-β-catenin and hedgehog pathways (Incardona and Eaton 2000), both of which are fundamental in the control of human hair follicle (HF) cycling (Lee and Tumbar 2012).

Dysregulation of cholesterol homeostasis has been implicated in numerous disorders of hair growth and cycling (Palmer et al. 2020). Briefly, mutations in the cholesterol biosynthetic pathway are associated with both patchy alopecia in Conradi–Hünermann syndrome (Braverman et al. 1999) and autosomal-recessive hypotrichosis simplex (Romano et al. 2018). Mutations to membrane-bound transcription factor peptidase site 2 (MBTPS2), which facilitates the cleavage of sterol regulatory element-binding protein 2 (SREBP2), are involved with ichthyosis follicularis, alopecia and photophobia (IFAP) (Jiang et al. 2019) as well as keratosis follicularis spinulosa decalvans (KFSD) (Zhang et al. 2016), resulting in non-progressive alopecia and cicatricial alopecia, respectively. Furthermore, mutations in the putative cholesterol transporter ATP-binding cassette (ABC) subfamily A member 5 (ABCA5) are associated with a form of congenital hypertrichosis (DeStefano et al. 2014; Hayashi et al. 2017).

Cholesterol homeostasis is tightly regulated and coordinated by a host of transcription factors, including liver X receptor (LXR) and peroxisome proliferator-activated receptor gamma (PPARγ). In cholesterol-rich environments, both are activated, resulting in increased cholesterol efflux via ABC transporters (Nakaya et al. 2011; Zhao and Dahlman-Wright 2010). Activation of LXR by the synthetic agonist T0901317 also reduces hair growth, suggesting an important link between the regulation of cellular cholesterol levels and hair biology (Russell et al. 2007). In addition, dysregulation of PPAR signalling is implicated in hair growth disorders such as primary cicatricial alopecias, following the downregulation of cholesterol synthesis (Karnik et al. 2009). Disruption of this synthetic pathway in murine models is associated with altered HF morphogenesis and progressive hair loss (Evers et al. 2010; Zhang et al. 2017).

Given the accumulated evidence supporting a role for cholesterol homeostasis in hair biology, alongside the established roles for cholesterol transport in this homeostatic process, we have examined the expression and localisation of critical cholesterol transport proteins in the human HF. Importantly, we have mapped their expression across the hair cycle, providing an insight into their relative abundance and expression patterns during the distinct stages of the hair cycle. Furthermore, the expression and activity of these proteins in response to LXR activation has been examined, demonstrating the capacity of the HF to regulate cholesterol homeostasis through activation of established signalling pathways.

## Methods

### Human tissue

Human scalp skin was obtained via surplus material from facelift surgery (Caltag Medsystems Ltd, Buckingham, UK), dissected and immediately frozen in Richard-Allan Scientific™ Neg-50™ Frozen Section Medium (Thermo Scientific™, Massachusetts, USA). Plucked human telogen HFs were obtained from the occipital or temporal scalp, and immediately frozen. All tissue was obtained with informed consent following ethical approval by the School of Applied Sciences Research Integrity and Ethics Committee (SRIEC) and in line with the University of Huddersfield Research Ethics and Integrity Policy and Code of Practice for Research. Tissue was cryosectioned at 7 μm. For experiments involving LXR agonism, HFs were cultured in Williams E medium (Gibco™, Massachusetts, USA) supplemented with 1% (100 IU and 100 µg/ml) penicillin/streptomycin (Lonza, Basel, Switzerland), 2 mM L-glutamine (Lonza), 10 µg/ml insulin (Sigma-Aldrich, Dorset, UK), 10 ng/ml hydrocortisone (Acros Organics, Geel, Belgium), as previously described (Langan et al. 2015; Philpott et al. 1989; Philpott 2018). HF staging was performed as described in (Kloepper et al. 2010; Oh et al. 2016). Donor ages are provided in Table S1.

### Cell culture

Outer root sheath (ORS) keratinocytes were isolated from plucked HFs, obtained from consenting donors. Briefly, cells were incubated in 0.25% Trypsin–EDTA (Gibco™) and agitated periodically, as previously described (Aasen and Izpisua Belmonte 2010; Bodo et al. 2005; Haslam et al. 2017; Limat and Noser 1986). Cells were seeded onto mitomycin C treated human dermal fibroblast feeder layers until confluent. Initial isolation media consisted of DMEM (Sigma-Aldrich) 3:1 Ham’s F12 (Lonza) supplemented with 10% fetal bovine serum (Gibco™), 2 mM L-glutamine, 0.4 µg/mL hydrocortisone (Acros Organics), 5 µg/mL insulin, 2.4 µg/mL adenine (Alfa Aesar™), 2 nM triiodothyronine (Sigma-Aldrich), 0.1 nM cholera toxin (Sigma-Aldrich), 10 ng/mL epidermal growth factor (Sigma-Aldrich), 1 mM ascorbyl-2-phosphate (Sigma-Aldrich), 100 UI/mL penicillin G (Alfa Aesar™) and 25 µg/mL gentamycin (Gibco™). Subculture was achieved through incubation with 1 × TrypLE Express (Gibco™) and subsequent growth performed in Epilife media (Gibco™) supplemented with human keratinocyte growth supplement (HKGS) (Gibco™).

### Immunofluorescence and cholesterol staining

All primary antibodies were purchased from Abcam (Cambridge, UK) except for HMGCR (Proteintech^®^, Manchester, UK) and CD200 (Bio-Rad, California, USA). Tissue sections were fixed and blocked as described in Table S2. Washes were performed with PBS (Fisher BioReagents™, Leicestershire, UK) or TBS (Alfa Aesar™) containing 0.1% tween 20. Incubation with primary antibodies was performed overnight at 4 °C. ABCA1 (1:25; ab18180), ABCA5 (1:200; ab99953), ABCG1 (1:50; ab52617), SCARB1 (1:200; ab217318), HMGCR (1:200, 13533-1-AP), CD200 (1:200; MCA1960GA), laminin-332 (1:1000; ab78286). Subsequently, slides were washed and incubated with secondary antibodies: Goat-anti-Rabbit Alexa Fluor^®^ 594 (1:200 for ABCA5 and HMGCR, 1:1000 for SCARB1; A11012, Invitrogen™, Massachusetts, USA), Goat-anti-Mouse Alexa Fluor^®^ 488 (1:200 for CD200 and laminin-332; A11001, Invitrogen™) or Goat-anti-Mouse Alexa Fluor^®^ 568 (1:200 for laminin-332; ab175701, abcam) for 45 min at room temperature. Alternatively, VectaFluor™ Excel Amplified DyLight^®^ 488 Anti-Mouse IgG Kit (for ABCA1; DK-2488, Vector^®^ Laboratories, Peterborough, UK), VectaFluor™ Excel Amplified DyLight^®^ 488 Anti-rabbit IgG Kit (for ABCG1; DK-1488, Vector® Laboratories) were used as per manufactures instructions. Nuclear co-localisation was achieved with 1 µg/mL DAPI (4′,6-ediamidino-2-phenylindole). For more details see Table S2. Filipin (Sigma-Aldrich) staining for free cholesterol was performed subsequent to fixation in 4% paraformaldehyde (PFA) for 10 min, quenched with 10 mg/ml glycine (Fisher) and filipin added at 100 µg/ml in 1 × PBS with 10% FBS for 2 h at room temperature.

### Fluorescence microscopy

Confocal microscopy was performed using Zeiss laser scanning microscope 880 Axio-Observer using Zen Black v2.3 software for acquisition (Zeiss). For HF sections, tile scan images were performed with Plan-Aprochromat 40x/1.4 Oil DIC M27 objective with a 10% overlap between tiles. For all images the following settings were used: digital gain 1.0, detector offset 0, binning 1 × 1 and averaging 1. Scaling of either 0.04 × 0.04 µm or 0.07 × 0.07 µm per pixel was used. Diode 405 nm, 488 nm line of Argon 458/488/514 nm, Diode 561 nm and HeNe 594 nm lasers were used at a range of 1–3%, detector gain setting varied from 600–800. Images were acquired using a 34-channel detector comprised of two high sensitivity red and blue PMT confocal detectors and a 32 channel GaAsP spectral detector. Airyscan processing was performed in Zen Black software. Zen Blue v2.3 was utilised for image stitching; histogram image processing and gamma correction were applied to all images universally. Regions of interests were selected from the tile scan images, scale bars donate 50 or 5 µm as shown in figure legends.

### Western blotting

For protein extraction, ORS keratinocytes were scraped in ice-cold RIPA buffer containing protease inhibitors (Pierce™, Massachusetts, USA) and sonicated for 30 s using a probe sonicator. Suspensions were centrifuged for 20 min at 12,000 rpm (4 °C), and supernatants were removed. 40–100 μg of total protein was loaded into Tris–Acetate gels (Invitrogen™) and run for 1 h at 150 V. Transfer was performed on iBlot2 (Invitrogen™) using PVDF membrane transfer stacks (Invitrogen™) for 10 min. Membranes were incubated in Intercept^®^ TBS blocking buffer (Licor^®^, Nebraska, USA) for 1 h and primary antibodies were incubated overnight at 4 °C on a rocker. ABCA1, ABCA5, ABCG1 (1:500), SCARB1 (1:1000), containing β-Actin (1:40,000 mouse; MAB1501, Millipore (U.K.) Limited, Hertfordshire, UK or rabbit; ab8227, Abcam). Membranes were washed with TBS-T then incubated with secondary antibodies at 1:1000 dilution, Goat anti-Mouse Alexa Fluor^®^ 680 (A21057, Invitrogen™), Goat anti-Mouse Alexa Fluor^®^ 790 (A11375, Invitrogen™) or Goat anti-Rabbit Alexa Fluor^®^ 680 (A21076, Invitrogen™) for 1 h. Membranes were imaged using Odyssey imaging system (Licor^®^). Densitometry was performed using image studio lite (Licor^®^).

### Cholesterol efflux assay

ORS keratinocytes were seeded at 2 × 10^4^ cells per well in a 48 well plate and incubated overnight. Cells were treated with 5 µM T0901317 or DMSO vehicle control (0.05%) for 3 days. Cells were washed in Epilife prior to incubation with BODIPY-cholesterol (Avanti lipids, Alabama, USA) (25 µM complexed 1:10 with methyl-β-cyclodextrin (MβCD; Sigma-Aldrich) for 2 h in the dark. Cells were washed in Epilife and equilibrated for 24 h with Acyl-CoA:cholesterol acyltransferase (ACAT) inhibitor, 2 µg/ml Sandoz 58–035 (Sigma-Aldrich). Cells were washed in a 10 mM HBSS-HEPES solution (Lonza) and efflux was initiated by adding cholesterol acceptors, 10 µg/ml Apolipoprotein A-I (ApoA1; Sigma-Aldrich) or 25 µg/ml high-density lipoprotein (HDL; Sigma-Aldrich) with 5 µM PSC833 (Sigma-Aldrich), 10 µM BLT-1 (Sigma-Aldrich) or DMSO vehicle control (0.18%) for 4 h. Background efflux was performed in the absence of cholesterol acceptors, with or without PSC833 or BLT-1. Media was removed and fluorescence measurements performed in black-walled 96 well plates (excitation 485/20 nm, emission 528/20 nm).

Time 0 monolayers were incubated with 1% cholic acid (Sigma-Aldrich) for 1 h at room temperature on a plate shaker. Cells were scraped and fluorescence measured (excitation 485/20 nm, emission 520/20 nm). Efflux calculations were performed, as previously described (Sankaranarayanan et al. 2011).

### Gene expression analysis

ORS keratinocytes were seeded into 12 well plates at 20 × 10^4^ cells per well and cultured overnight. For matching experiments, 10 HFs per treatment were incubated overnight in 24 well plates. Following this, both cells and HFs were incubated with 5 μM T0901317 or DMSO vehicle control (0.05%) (Sigma-Aldrich) for 24 h. ORS keratinocytes were pelleted prior to RNA extraction and HFs placed into RNAlater™ Stabilization Solution (Invitrogen™).

HF RNA extraction was performed using RNeasy micro Plus (QIAGEN, Hilden, Germany) following manufacturers guidelines. RNA extraction from ORS keratinocytes was performed using Relia prep (Promega, Wisconsin, United States) according to the manufacturer’s instructions. cDNA synthesis was achieved using Tetro cDNA synthesis kit (Bioline, London, UK) converting 100–1000 ng of RNA.

qPCR was performed using TaqMan™ gene expression assays (Applied biosciences™, Massachusetts, USA) and Precision Fast mastermix (Primerdesign, Southampton, UK) with a StepOne plus instrument (Applied biosciences™). Analysis was performed using ΔΔCT method. Taqman assay IDs: ABCA1; Hs01059118_m1, ABCA5; Hs00363322_m1, ABCG1; Hs00245154_m1, PPIA; Hs99999904_m1, HMGCR; Hs00168352_m1, SCARB1; Hs00969821_m1.

### Statistical analysis

Statistical analysis was performed using Prism (GraphPad, California, USA). Normality testing via Shapiro–Wilk testing was performed prior to two-tailed, one-sample *t*-tests, unpaired t-tests or one-way ANOVA with Dunnett’s comparisons, as appropriate. Statistical significance was described as *p ≤ 0.05.

## Results

Fig. S1 provides a schematic representation of the hair cycle (see supplementary material) and morphology of the cell layers in a sagittal HF tissue section.

### The cholesterol efflux transporter, ABCA1, is highly expressed in the human hair follicle

**Fig. 1:**
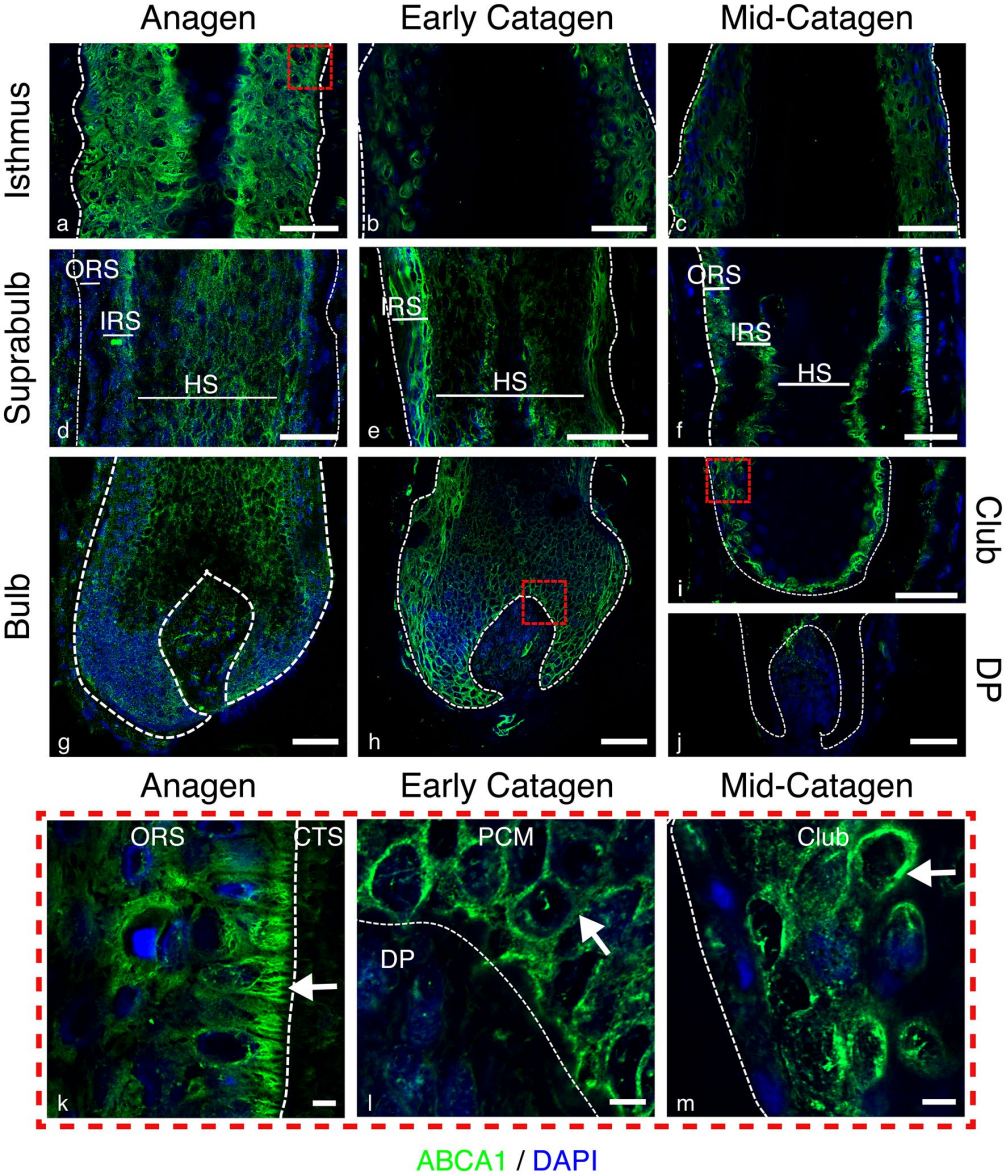
Localisation of ABCA1 (green) immunofluorescence staining in human anagen, early catagen and mid-catagen HFs with DAPI counterstaining (blue). Membrane ABCA1 expression is present in the matrix of anagen (**g**) and early catagen (**h**). Anagen magnified image (**k**), white arrow demonstrates polarity of ABCA1 expression to the membrane of the basal layer of the ORS within the isthmus. Membrane expression pattern is shown in early catagen magnified image as represented by the white arrow (**l**). Perinuclear pattern is found within the club hair of the mid-catagen follicle (**i**), as shown by the white arrow in the magnified image (**m**). Dashed white lines showing epithelial components, scale bars 50 μm. Red dashed box delineates magnified images, scale bar represents 5 μm. ORS, outer root sheath; IRS, inner root sheath; HS, hair shaft; CTS, connective tissue sheath; PCM, pre-cortical matrix; DP, dermal papilla; M, matrix. Representative images from *N* = 3 donors, with imaging performed in 2–3 follicles per donor (except mid-catagen, *N* = 2 donors, with 2 follicles per donor imaged)

The distribution of the ubiquitous cholesterol efflux transporter, ABCA1, was examined across the hair cycle. Anagen HFs displayed a distinct staining pattern, showing higher expression in the epithelial compartment compared with the mesenchymal connective tissue sheath (CTS) (Fig. 1a, d, g). Within the CTS and the dermal papilla (DP), staining was predominantly cytoplasmic, with some indication of increased perinuclear staining in the DP (Fig. 1g). The elongated staining pattern within the mesenchymal compartment suggested possible expression in the endothelium. The outer root sheath (ORS) of the isthmus displayed the highest staining intensity (Fig. 1a), with polarised expression in the basal ORS (Fig. 1k). Membrane staining was apparent in the pre-cortical matrix (PCM) and matrix keratinocytes (Fig. 1g). Whilst staining was indistinct within the inner root sheath (IRS), membranous localisation was apparent in the hair shaft (HS) cuticle (Fig. 1d).

Similar staining patterns were observed in early catagen, with distinct membrane staining in the matrix and PCM (Fig. 1h, l). The CTS and DP (Fig. 1h) showed a lower intensity of staining, compared with the suprabulbar IRS (Fig. 1e). Membrane staining was observed throughout the ORS and IRS. In mid-catagen, higher expression was found in the developing club hair (Fig. 1i), with perinuclear staining (Fig. 1m), and no expression was in the CTS or DP (Fig. 1j). Beyond the staining shown for anagen, early catagen and mid-catagen HFs (Fig. 1), staining was also examined in club hairs from plucked telogen HFs (Fig. 7). High expression of ABCA1 was only found within the inner bulge layers, with a predominantly perinuclear localisation (Fig. 7b).

### ABCG1 is highly expressed in the sebaceous gland

**Fig. 2:**
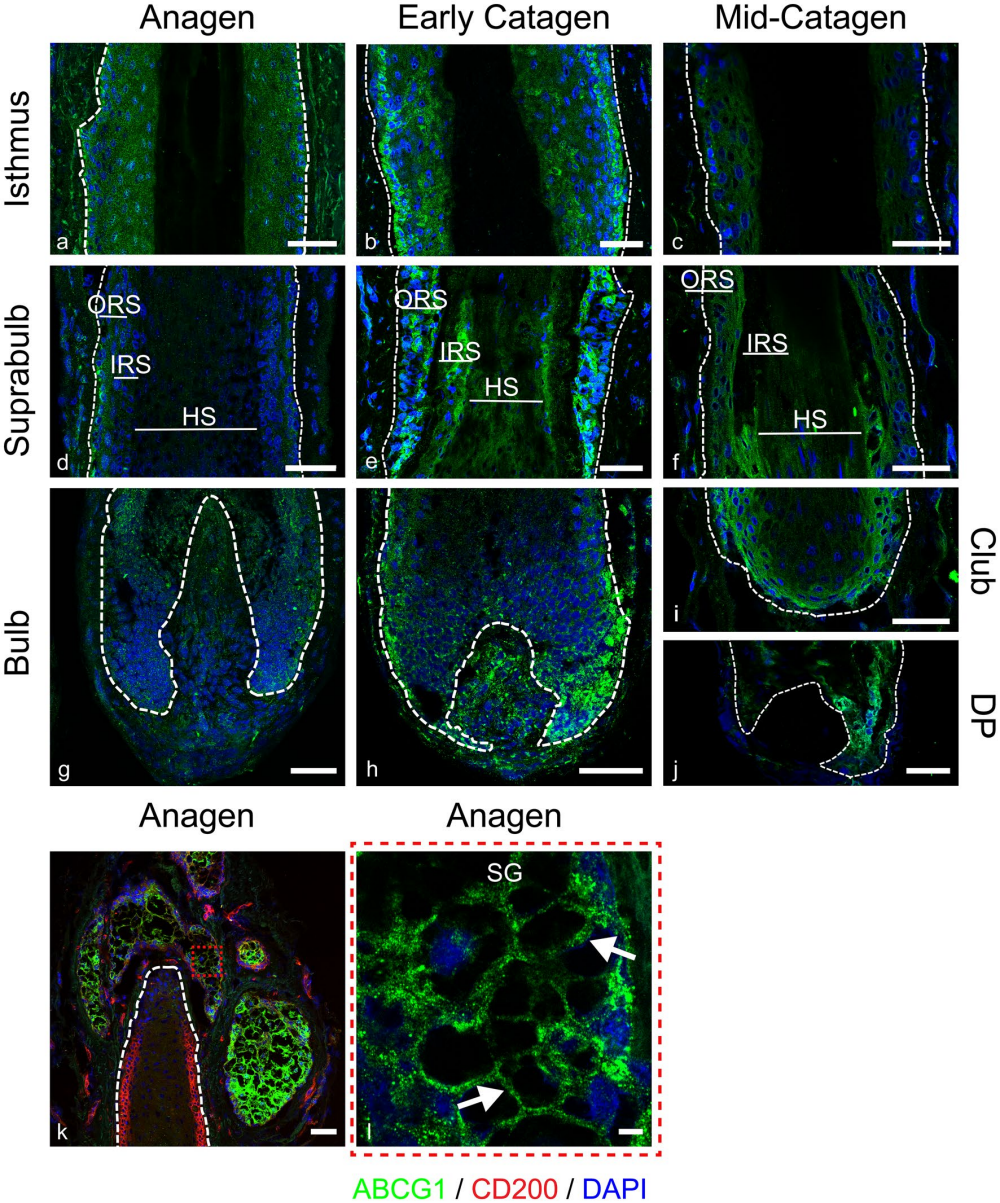
ABCG1 expression is present in the membrane of the sebaceous gland, albeit low in the HF. ABCG1 immunofluorescence (green) staining in anagen (**a**, **d**, **g**, **k**, **l**), early catagen (**b**, **e**, **h**) and mid-catagen (**c**, **f**, **i**, **j**) HFs with DAPI counterstaining (blue). Dashed white lines showing epithelial components, scale bars 50 μm, Red dashed box delineates magnified images, scale bar 5 μm. ABCG1 is highly expressed in the sebaceous gland, with very low levels shown in the CD200 positive bulge region of the HF (**k**). Membrane staining in the sebaceous gland as shown in the magnified image by the white arrow (**l**). HF images intensity adjusted to represent the localisation patterns of the weekly expressed ABCG1. ORS; outer root sheath, IRS; inner root sheath, HS; hair shaft, SG; sebaceous gland. Representative images from *N* = 3 donors, with imaging performed in 2–3 follicles per donor (except mid-catagen, *N* = 2 donors, with 2 follicles per donor imaged)

In addition to ABCA1, the physiologically important cholesterol transporter ABCG1 was also examined (Fig. 2). Immunofluorescence detection of ABCG1 was found to be low throughout the HF, however, considerably higher staining levels were seen in the sebaceous gland (Fig. 2k, i).

**Fig. 3:**
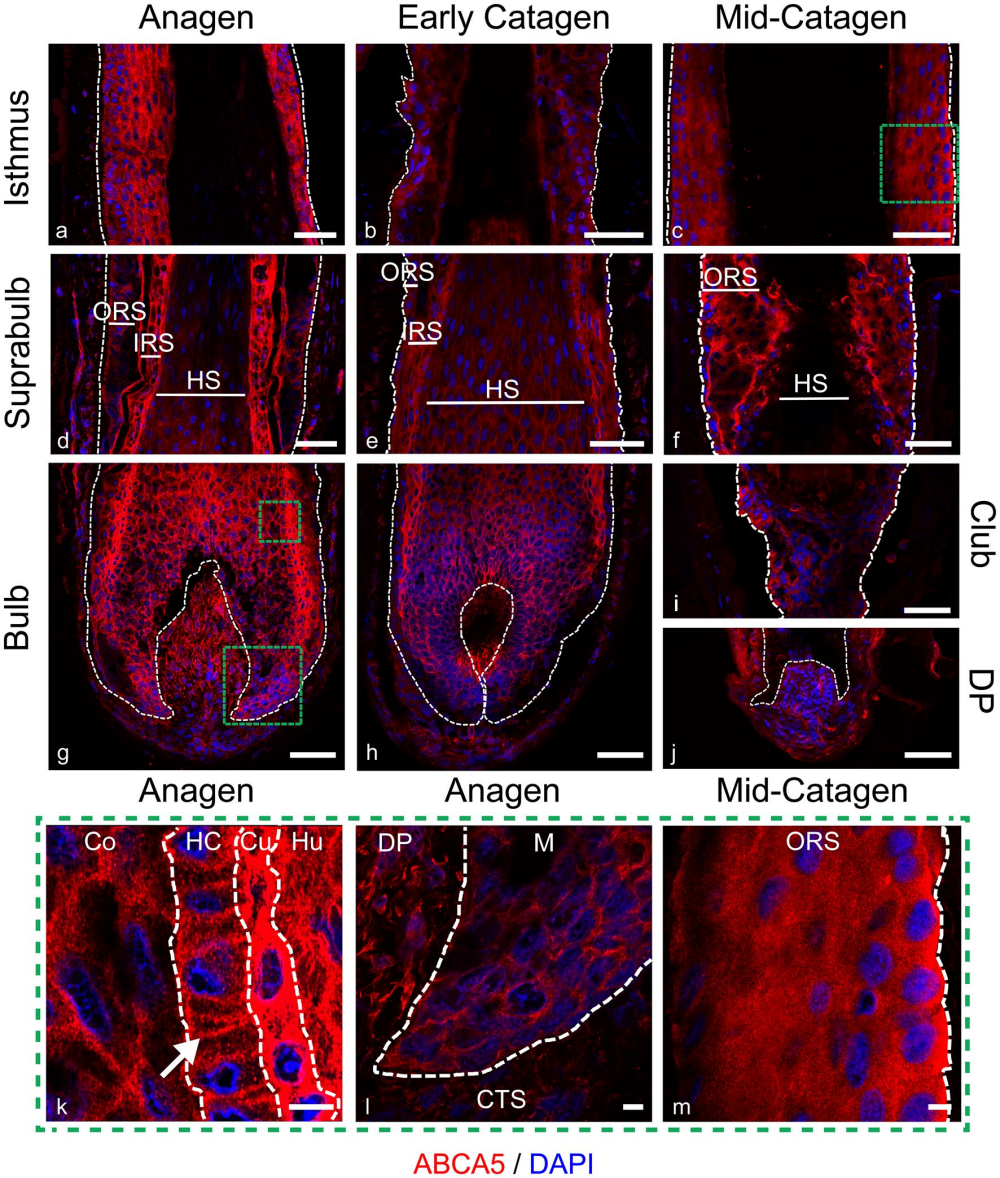
ABCA5 protein levels are most highly expressed in the IRS ABCA5 (red) immunofluorescence staining in anagen (**a**, **d**, **g**, **k**, **l**), early catagen (**b**, **e**, **h**) and mid-catagen (**c**, **f**, **i**, **j**, **m**) HFs with DAPI counterstaining (blue). Dashed white lines showing epithelial components. Scale bars 50 μm, magnified images represented by the green dashed box, scale bar 5 μm. Magnified IRS and HS of anagen follicle from the bulb region showing membrane staining in the HS cuticle demonstrated by the white arrow (**k**). Presence of cytoplasmic ABCA5 in both the DP and matrix keratinocytes (**i**). Polar staining towards the basal membrane of the basal layer of ORS keratinocytes in the mid-catagen follicle as shown by the white arrow (**m**). ORS; outer root sheath, IRS; inner root sheath, HS; hair shaft, CTS; connective tissue sheath, DP; dermal papilla, M; matrix, Co; cortex, HC; hair cuticle, Cu; cuticle, Hu; Huxley’s layer, SG; sebaceous gland. Representative images from *N* = 3 donors, with imaging performed in 2–3 follicles per donor (except mid-catagen, *N* = 2 donors, with 2 follicles per donor imaged)

The highest levels of ABCG1 staining was seen within the ORS of anagen HFs (Fig. 2a, d), a pattern also observed during early catagen (Fig. 3b, e). Little expression was seen within the matrix keratinocytes, PCM and ORS (Fig. 2h) in anagen.

Mid-catagen HFs showed a broadly comparable staining pattern (Fig. 2c, f), however, staining intensity increased in the IRS proximal to the club hair, with the club hair itself showing more intense staining (Fig. 2i). Low levels of ABCG1 were found within the mesenchymal compartments of the HF across the hair cycle (Fig. 2). Within the plucked telogen HF staining was largely indistinct although notably, a higher intensity of staining at the tip of the club hair was observed consistently across HFs from three individual donors (Fig. 7f).

### ABCA5 is highly expressed in the inner root sheath and membranous in the hair shaft cuticle

Next, expression and localisation of putative cholesterol transporter ABCA5 was examined across the hair cycle (Fig. 3). As previously reported (DeStefano et al. 2014), expression was high in both the ORS and IRS of anagen HFs (Fig. 3a, d, g), with the suprabulbar ORS showing lower expression than the ORS of the isthmus (Fig. 3a). In the mesenchymal regions of the HF expression was high in the DP, yet substantially lower in the CTS (Fig. 3g). Both the matrix (Fig. 3l) and PCM displayed high-intensity ABCA5 staining. Within anagen HFs, sub-cellular expression was predominately cytoplasmic, however, membrane staining is apparent within the HS cuticle (HC [Fig. 3k]). The highest intensity staining was shown the IRS cuticle (Cu), with the IRS and the companion layer showing the greatest intensity ABCA5 staining in suprabulbar regions (Fig. 3d).

ABCA5 staining patterns in early catagen were largely consistent with that of anagen HFs, though staining intensity within the matrix and ORS was somewhat lower (Fig. 3h).

By contrast, mid-catagen HFs showed much lower levels of ABCA5 (Fig. 3 c,f,i). Staining intensity in the IRS was extremely low and was undetectable in the HS (Fig. 3f). In opposition to this, high-intensity staining was apparent in the DP and the CTS in proximity to this area (Fig. 3j). Staining was polarised to the basal layer of the ORS (Fig. 3m) of the isthmus.

Within plucked telogen HFs (Fig. 7c), staining polarity showed higher expression levels in the ORS cells adjacent to the basement membrane, with a largely cytoplasmic pattern (Fig. 7d).

### Distinct SCARB1 staining is found in the dermal papilla basement membrane

**Fig. 4:**
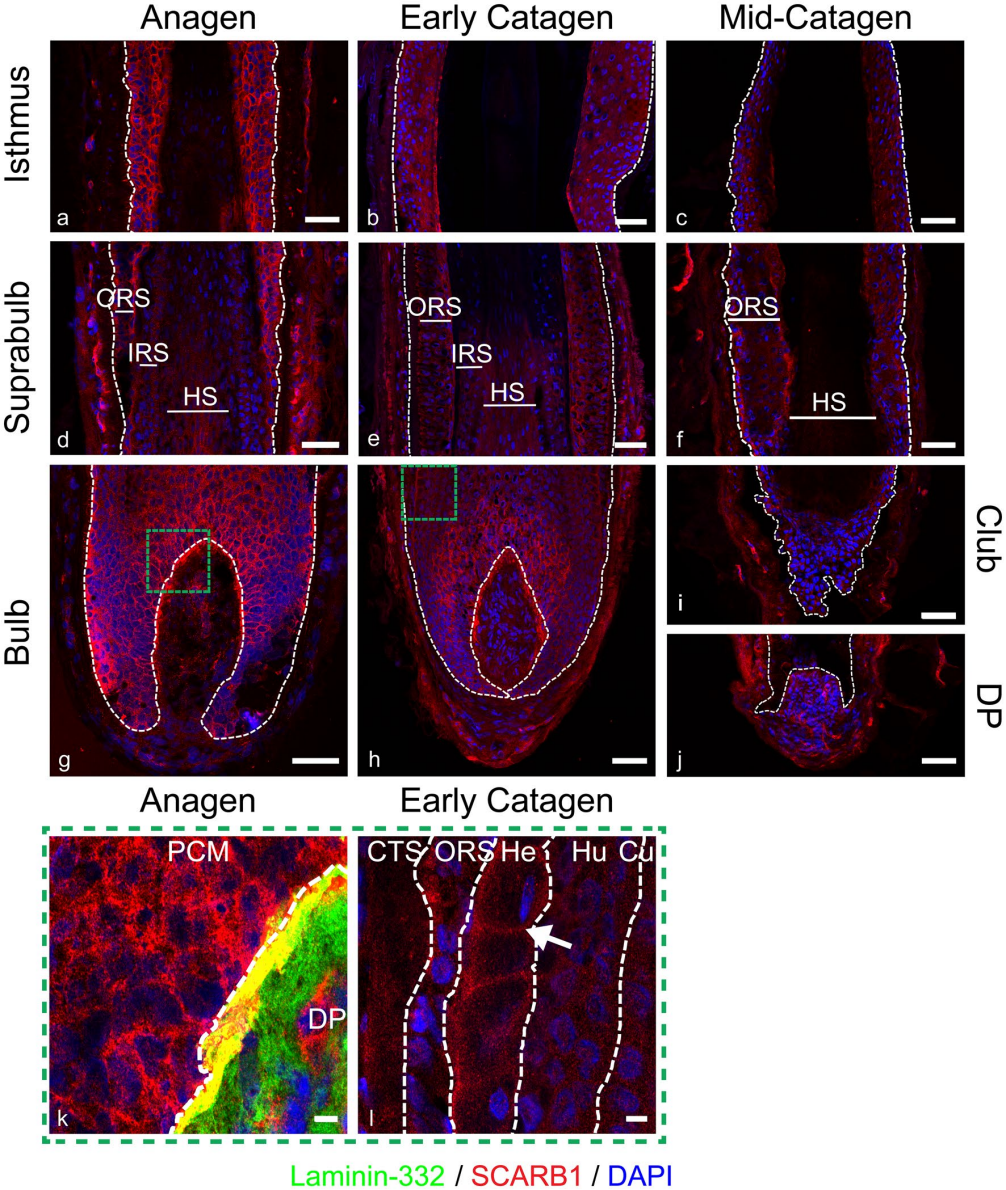
SCARB1 is present in the DP basement membrane. SCARB1 immunofluorescence staining (red) in anagen (**a**, **d**, **g**, **k**), early catagen (**b**, **e**, **h**, **l**) and mid-catagen (**c**, **f**, **i**, **j**) HFs with DAPI counterstain (blue). Dashed white lines showing epithelial components, scale bars 50 μm. Green dashed box delineates magnified images, scale bar 5 μm. Magnified image of DP co-stained with laminin-332 (green) showing co-localisation of SCARB1 to laminin-332 as represented by the white arrow (**k**). Membrane staining of SCARB1 in Henle’s layer of the IRS within early catagen follicle (**l** white arrow). ORS; outer root sheath, IRS; inner root sheath, HS; hair shaft, DP; dermal papilla, M; matrix, Cu; cuticle, Hu; Huxley’s layer, He; Henle’s layer. Representative images from *N* = 3 donors, with imaging performed in 2–3 follicles per donor (except mid-catagen, *N* = 2 donors, with 2 follicles per donor imaged)

In addition to the ABC transporters examined above, the expression pattern of the bi-directional HDL transporter SCARB1 was investigated, given documented evidence describing its influence on cholesterol homeostasis in numerous tissues (Shen et al. 2018a, b; Sticozzi et al. 2012) (Fig. 4). Staining intensity in anagen HFs was high within the bulb, with membrane staining apparent throughout (Fig. 4g). Mesenchymal immunofluorescence was low, with some higher intensity elongated staining patterns that could indicate expression in the HF vasculature.

To investigate the distinct halo of SCARB1 expression observed surrounding the DP, dual staining with the basement membrane marker laminin-332 was performed. As shown in Fig. 4k, a clear co-localisation of SCARB1 with laminin-332 was seen. The antibody used to detect SCARB1 recognises the extracellular domain, which would face the DP basement membrane (Shen et al. 2018b). This may suggest a role for SCARB1 in facilitating the movement of cholesterol between the DP and matrix keratinocytes.

In the early catagen HFs, membrane staining was present in Henle’s layer of the IRS (Fig. 4l). Membrane staining continues in the suprabulbar ORS (Fig. 4e). As seen with ABCG1, SCARB1 expression was lower in mid-catagen HFs (Fig. 4c, f, i, j) in comparison with early catagen or anagen.

During telogen, distinct membrane staining was found (Fig. 7i–k), with some co-localisation to the CD200 expressing stem cells found in the basal and lateral membranes (Fig. 7k).

### Cholesterol synthesis enzyme, HMGCR, is highly expressed throughout the hair cycle

**Fig. 5:**
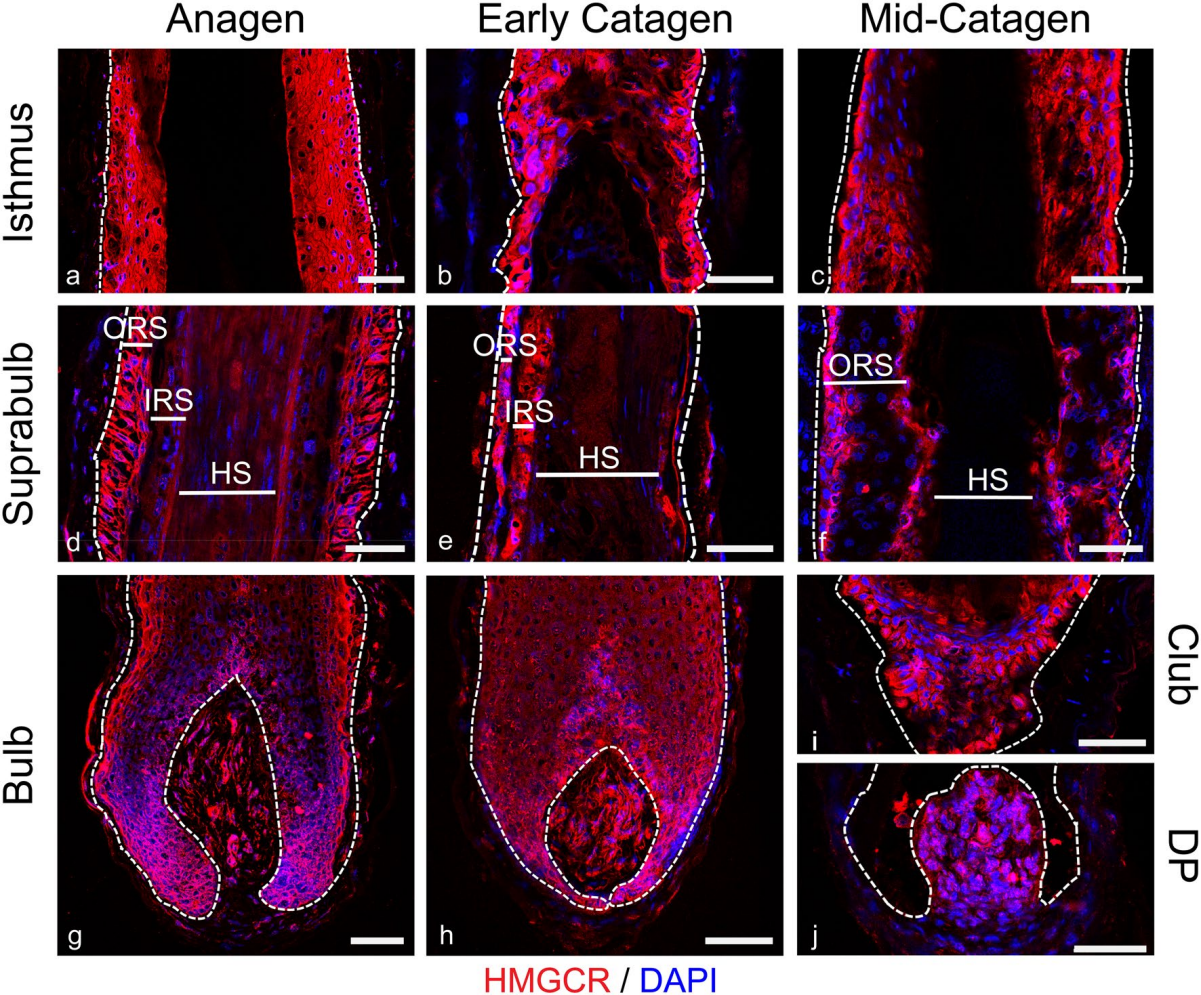
HMGCR is present throughout the hair cycle, high expression is seen in the ORS, Matrix and DP. HMGCR immunofluorescence staining (red) in anagen (**a**, **d**, **g**), early catagen (**b**, **e**, **h**) and mid-catagen (**c**, **f**, **i**, **j**) HFs with DAPI counterstaining (blue). Dashed white lines showing epithelial components. Scale bars 50 μm. ORS; outer root sheath, IRS; inner root sheath, HS; hair shaft. Representative images from *N* = 3 donors, with imaging performed in 2–3 follicles per donor (except mid-catagen, *N* = 2 donors, with 2 follicles per donor imaged)

Further to the expression of cholesterol transporters, cholesterol handling in the HF was investigated through localisation of 3-hydroxy-3-methylglutaryl-coenzyme A reductase (HMGCR), the enzyme responsible for the rate-limiting step in the biosynthetic pathway (Fig. 5). During anagen (Fig. 5a, d, g), intense staining was found in the matrix, DP and ORS (being highest within the isthmus) with lower levels in the IRS and HS. Staining in the CTS was low to absent. During early catagen (Fig. 5b, e, h) the intensity of HMGCR staining in the epithelial HF was lower than in anagen, though remained high in the DP. During mid-catagen, high expression was seen in the epithelial strand and developing club hair (Fig. 5i), as well as the ORS of the isthmus (Fig. 5c). Within the plucked telogen HF (Fig. 7l) the highest intensity of staining was found within the medial layers of the inner bulge. The widespread expression of HMGCR across the hair cycle points to the HFs capability for de novo cholesterol synthesis.

### Filipin staining identifies distinct membrane cholesterol staining across the hair cycle with striations present in the basement membrane

**Fig. 6:**
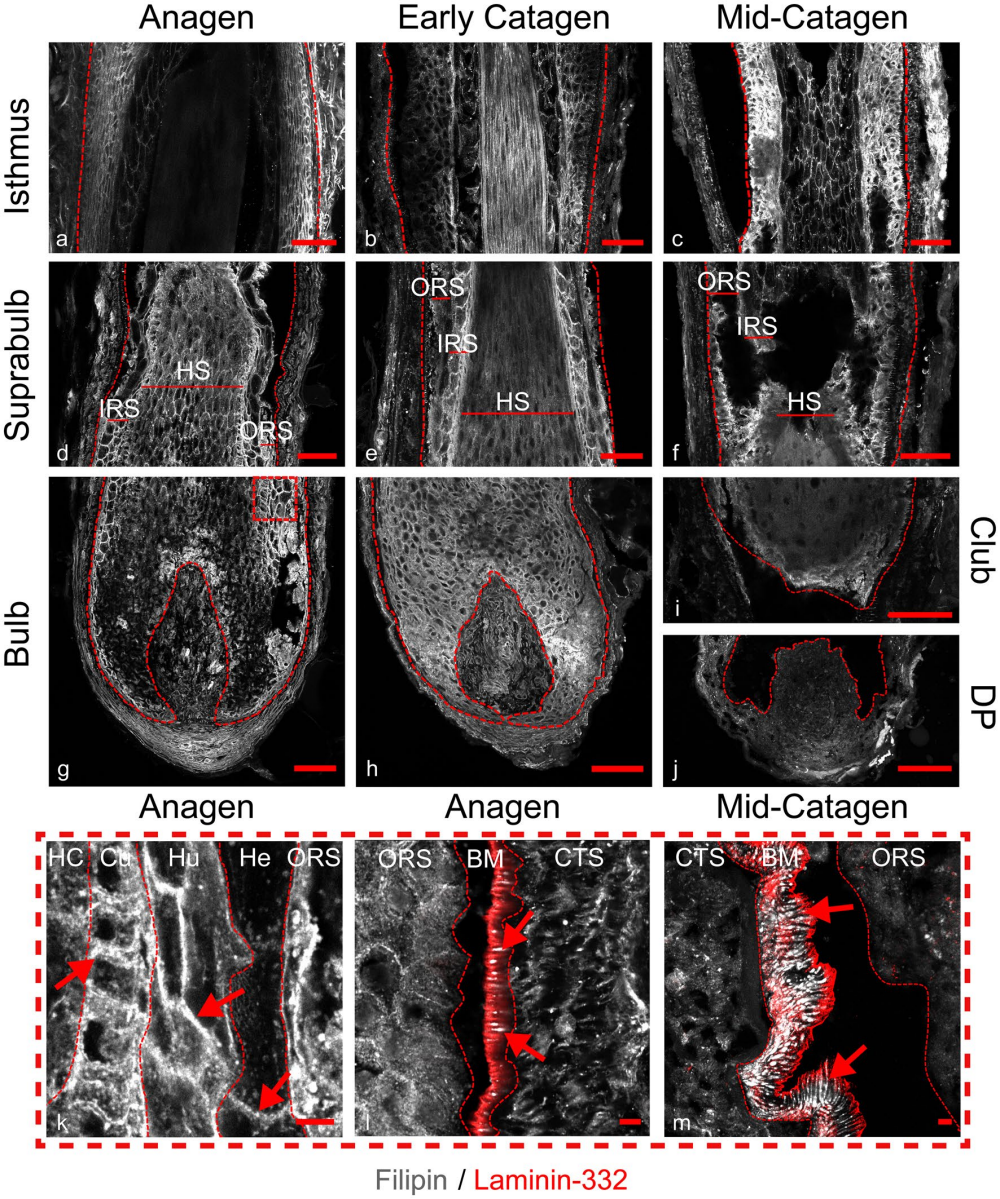
Differential expression of cholesterol throughout the hair cycle. Filipin immunofluorescence staining (greyscale) in HF tissue sections of anagen (**a**, **d**, **g**), early catagen (**b**, **e**, **h**) and mid-catagen (**c**, **f**, **i**, **j**) HFs. Red dashed lines showing epithelial components, scale bars 50 µm. Magnified images represented by the red dashed box, scale bars 5 μm. Membrane staining is present in the IRS of anagen follicle at the suprabulbar level as indicated by the red arrows (**k**). Striations of cholesterol within the basement membrane as shown by co-staining with laminin-332 (red) (**l**). Basement membrane is outlined with a red dashed line, striations indicated with red arrows. During mid-catagen these striations increase in both volume and intensity (**m**). ORS; outer root sheath, IRS; inner root sheath, HS; hair shaft, Co; cortex, HC; hair cuticle, Cu; cuticle, Hu; Huxley’s layer, He; Henle’s layer, ES; epithelial strand, BM; basement membrane. Representative images from *N* = 3 donors, with imaging performed in 2–3 follicles per donor (except mid-catagen, *N* = 2 donors, with 2 follicles per donor imaged)

Beyond examining the expression and localisation of proteins involved in cholesterol homeostasis, changes in free cholesterol distribution were investigated using filipin staining (Fig. 6). The antibiotic filipin iii is a naturally fluorescent probe, which binds to the 3-β-hydroxy group of sterols, making it selective in detecting free cholesterol and not cholesterol esters (Maxfield and Wüstner 2012). During anagen, mesenchymal filipin staining was somewhat diffuse compared with the distinct membrane staining pattern seen in the HF keratinocytes (Fig. 6a, d, g). Striations of filipin staining were present in the basement membrane of the ORS and CTS from the level of the suprabulbar region and continue into the isthmus but not the bulb (Fig. 6l, m).

To further investigate these striations, immunofluorescent staining of basement membrane protein laminin-332 was performed along with filipin staining. Images (Fig. S2) reveal that these cholesterol striations were present within the laminin-332 region. This is in contrast with the basement membrane of the epidermis, where no filipin-stained striations were observed (Fig. S2), suggesting this extracellular cholesterol is unique to the HF basement membrane distal to the bulb.

Within the ORS both membrane and cytoplasmic staining could be observed (Fig. 6l). The greatest intensity membrane staining was found within the membranes of the IRS (Fig. 6k), particularly in the cuticle and Huxley’s layer. Within the HS, both the cuticle and cortex had distinctive membrane staining. Free cholesterol was present throughout the CTS, particularly within the DP stalk (Fig. 6g).

A similar pattern of filipin staining was observed during early catagen (Fig. 6b, e, h). Notably, filipin staining within the matrix keratinocytes was of higher intensity than observed in the anagen HFs (Fig. 6h). During mid-catagen filipin levels in the ORS remained high (Fig. 6c). At the level of the club hair, filipin staining is notably lower and more diffuse (Fig. 6i).

**Fig. 7:**
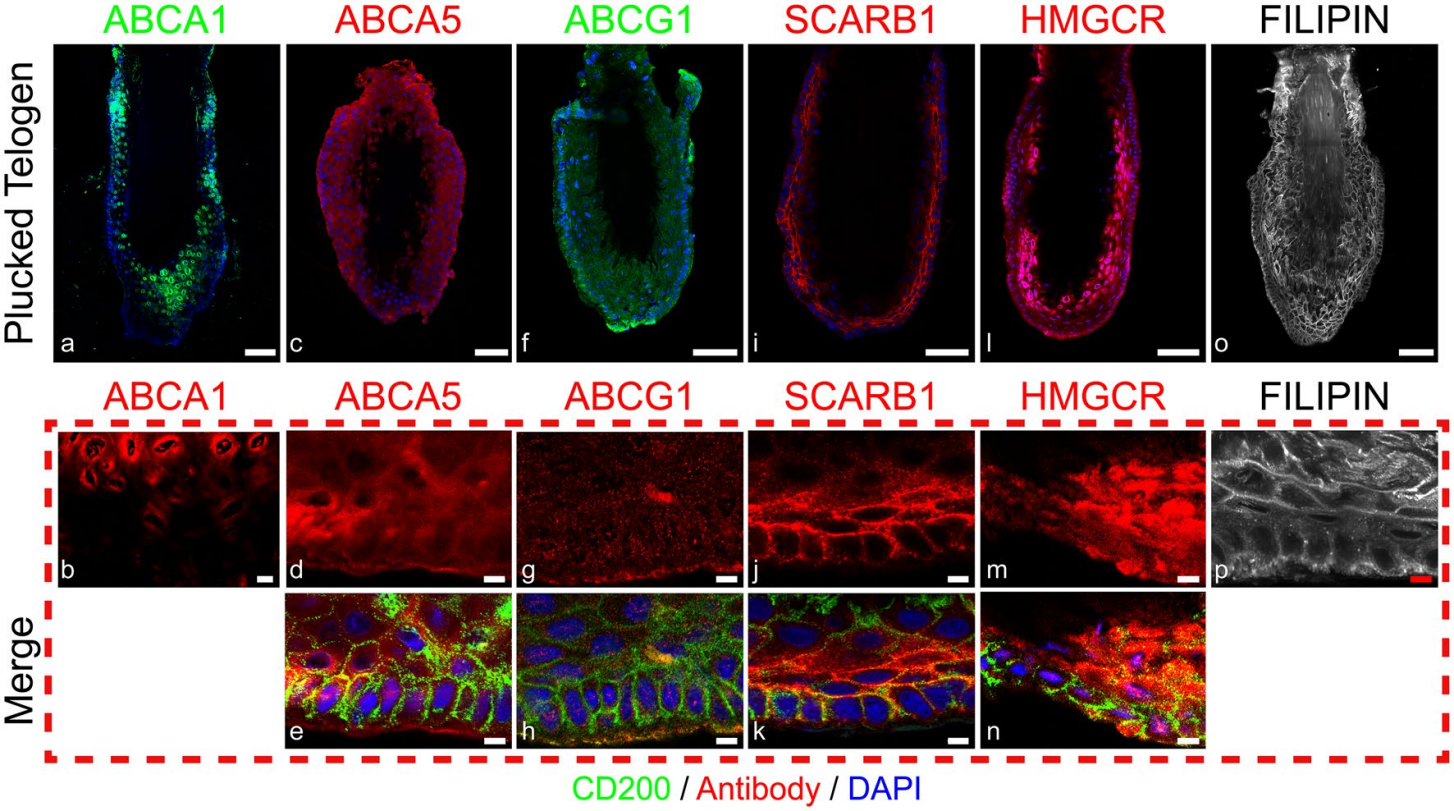
Cholesterol transporters are differentially located in plucked telogen HFs. Immunofluorescence staining of ABCA1 (**a**, **b**), ABCA5 (**c**–**e**), ABCG1 (**f**–**h**), SCARB1 (**i**–**k**) and HMGCR (**l**-**n**) (protein colour represented by legend) counterstained with DAPI (blue). Along with filipin staining (greyscale) for cholesterol (**o**, **p**). Scale bars 50 µm. Red dashed box represents magnified areas, scale bars 5 µm, co-localised with CD200 immunofluorescence staining (green). Perinuclear expression of ABCA1 found within the inner bulge layers (**b**). ABCA5 staining is largely cytoplasmic (**d**). Very low level of ABCG1 is seen (**g**), however, a small amount is found in the outer membrane with CD200 co-localisation (**h**), as shown by the white arrows. SCARB1 membrane staining is present within innermost layers of the inner bulge and the later membrane of the outer bulge (**j**). Cholesterol synthesis is present within the telogen follicle (**l**). Filipin staining is higher in the membranes of the inner bulge (**p**). Representative images representative from *N* = 3–4 donors, with imaging of *N* = 2 follicles per donor

Filipin staining in plucked telogen HFs showed that both layers of the bulge had a highly membranous staining pattern (Fig. 7o, p).

### Liver X Receptor agonism dynamically regulates cholesterol efflux in the hair follicle

Besides determining changes in protein distribution in the HF, changes in the expression of these cholesterol homeostatic genes were examined. Their regulation by LXR, an important transcription factor in the control of cholesterol homeostasis, was tested in HFs following a 24-h incubation with the agonist, T0901317.

**Fig. 8:**
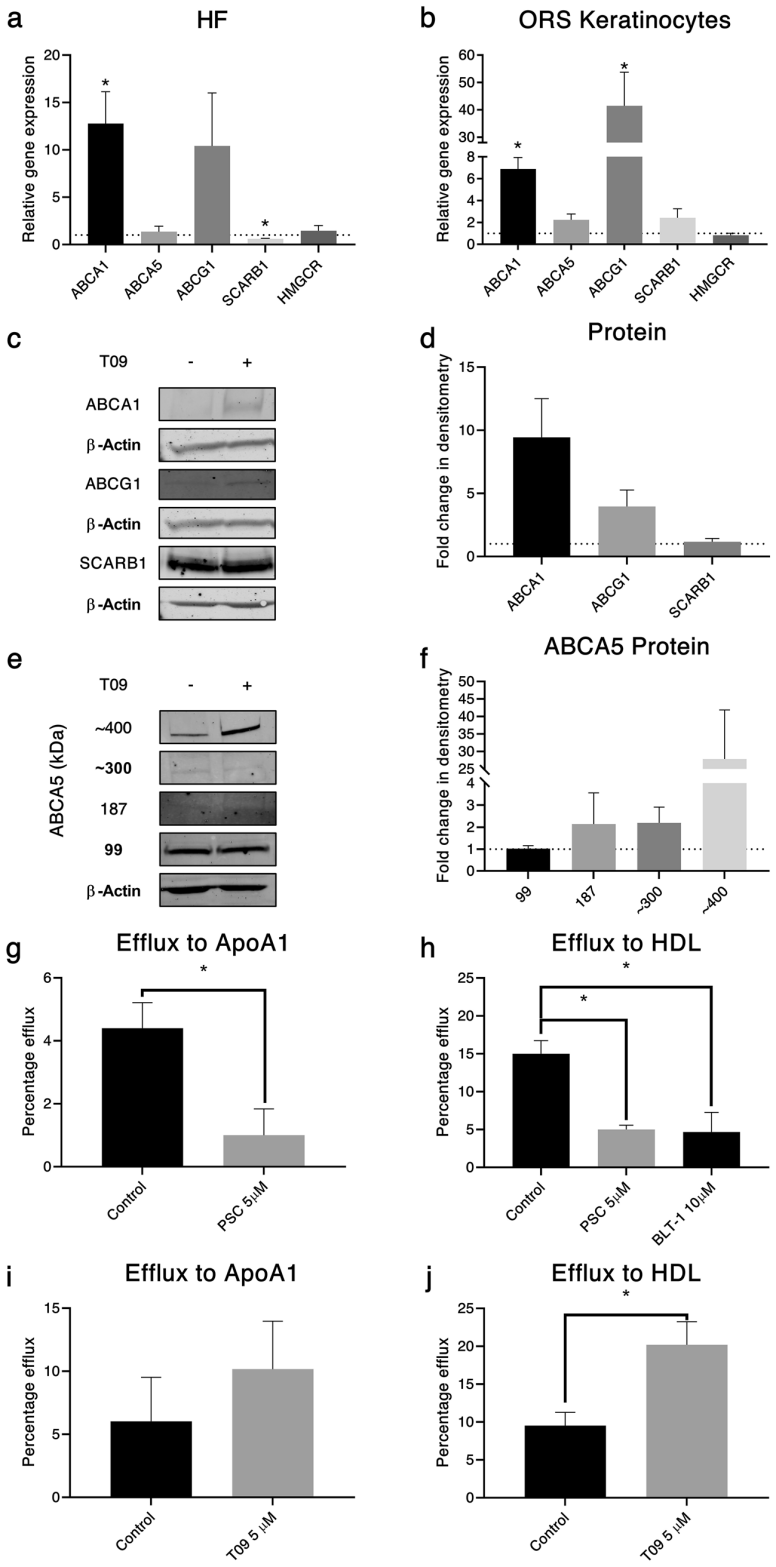
Cholesterol transporters are differentially expressed with LXR activation. (**a**) *ABCA1* mRNA expression increases in HFs treated with 5 µM T0901317 or DMSO control (0.05%) for 24 h (*N* = 4). (**b**) Gene expression of *ABCA1* and *ABCG1* increases with LXR activation in outer root sheath (ORS) keratinocytes treated with 5 µM T0901317 or DMSO control (0.05%) for 24 h (*N* = 4). Gene expression normalised to *PPIA* and vehicle control-treated HFs/ORS keratinocytes. (**c)** Protein expression of ABCA1 and ABCG1 increased with LXR activation. Representative western blot for ABCA1, ABCG1 and SCARB1 of ORS keratinocytes treated with 5 µM T0901317 or DMSO control (0.05%) for 72 h. (**d**) Protein densitometry of western blots in (**c**) (*N* = 4) normalised to β-actin and vehicle controls. (**e**) ABCA5 forms a 400 kDa tetramer, which protein level increases with LXR activation. Western blot of ABCA5 protein in ORS keratinocytes treated with 5 µM T0901317 for 72 h. Isoforms at 187 and 99 kDa along with oligomers of 300 and 400 kDa. (**f**) Protein densitometry of western blots in (**e**) (*N* = 4) normalised to β-actin and vehicle controls. Cholesterol efflux in ORS keratinocytes can be mediated by ABCA1, ABCG1 and SBR1. Percentage change in cholesterol efflux to 10 µg/ml ApoA1 (g, i) or 25 µg/ml HDL (h, j) in ORS keratinocytes pre-treated with 5 µM T0901317 or DMSO control (0.05%) for 72 h prior to 2-h loading with 25 µM BODIPY cholesterol or the presence of 5 µM PSC833, 10 µM BLT-1 or DMSO control (0.18%) during efflux compared to vehicle control (*N* = 6). One-sample *t*-test (a, b), unpaired *t*-test (g, i, j), one-way ANOVA (h) *p ≤ 0.05

The expression of *ABCA1* and *ABCG1* increased in response to LXR activation in HFs, whereas *SCARB1* was reduced (Fig. 8a). Subsequently, primary ORS keratinocytes were subject to the same treatment, showing significant upregulation of both *ABCA1* and *ABCG1* (Fig. 8b). A 72-h treatment of ORS keratinocytes with T0901317, followed by western blot analysis and densitometry, identified an increase in ABCA1 and ABCG1 protein levels, with no change in SCARB1 protein expression (Fig. 8c, d).

In probing the expression of ABCA5 by western blot, four isoforms were identified (Fig. 8e), the predicted 99 kDa and 187 kDa isoforms, along with two additional bands at approximately 300 and 400 kDa. Densitometry showed no change in the level of 99, 187 or 300 kDa isoforms, however, a large increase in the 400 kDa isoform was observed, albeit with substantial variability between donor ORS keratinocytes. To determine the oligomeric status of ABCA5 in protein lysate from untreated cells, the protein was run under non-denaturing/denaturing conditions, with and without heat (Fig. S3). This revealed that, in the absence of DTT and heat, only the 99 kDa and 400 kDa bands were present. These preliminary results may indicate the formation of ABCA5 trimers and tetramers, which will require further research to confirm.

Although defining the expression and localisation of proteins involved in cholesterol transport and homeostasis helps identify their potential roles in HF biology, it does not indicate functional capacity. As such, cholesterol transport activity was measured in primary ORS keratinocytes isolated from freshly plucked anagen HFs. Cells were incubated with BODIPY-cholesterol, and transport initiated in the presence or absence of cholesterol acceptors for ABCA1 (ApoA1) and ABCG1/SCARB1 (HDL). Non-specific ABC transporter inhibition using PSC833 resulted in a significant decrease in cholesterol efflux to both ApoA1 (Fig. 8g) and HDL (Fig. 8h). To examine whether SCARB1 participated in cholesterol movement in ORS keratinocytes, cholesterol efflux to HDL was determined in the presence of the specific SCARB1 inhibitor BLT-1. As SCARB1 cannot efflux cholesterol to ApoA1, and BLT-1 is not reported to inhibit efflux to ApoA1 (Nieland et al. 2004) the SCARB1 inhibitor was not used to examine efflux to this acceptor. A significant reduction in cholesterol efflux to HDL was seen (Fig. 8h), suggesting SCARB1, which is mostly reported to be involved in cholesterol uptake, is capable of cholesterol efflux in ORS keratinocytes. LXR agonism significantly increased efflux to HDL (Fig. 8j) but not ApoA1 (Fig. 8i). Basal cholesterol efflux to HDL was higher (15%) than efflux to ApoA1 (4.4%).

## Discussion

Here we describe the expression, localisation and regulation of transport proteins involved in cholesterol homeostasis across the human hair cycle. ABCA1 and ABCG1, two highly characterised proteins involved in modulating intracellular cholesterol levels, displayed both membranous and intracellular staining patterns as previously described (Neufeld et al. 2001). ABCA5, a putative cholesterol transporter implicated in a form of heritable hypertrichosis (DeStefano et al. 2014; Hayashi et al. 2017) also localised to both the plasma membrane and intracellular compartments. The bidirectional cholesterol transporter SCARB1 was highly expressed in the DP basement membrane, suggesting a possible role in modulating movement of sterols between the DP and matrix keratinocytes. Changes in expression across the hair cycle suggest that cholesterol transport and homeostasis is dynamically regulated, changing to meet cellular needs during different hair cycle phases. Furthermore, our data suggest that LXR activity plays a role in regulating cholesterol transporter expression and activity in HF cell populations.

Whereas the expression of ABCA1 in anagen HFs has been described previously (Haslam et al. 2015), here we expand this analysis to include catagen and telogen. Cellular localisation of ABCA1 is dependent on both HF region and hair cycle stage, with matrix keratinocytes of anagen and early catagen HFs showing plasma membrane staining, whereas perinuclear expression is apparent within the isthmus of catagen HFs and telogen club hairs. These changes in the subcellular localisation of ABCA1 would be expected to alter cholesterol efflux capacity. Although ABCA1 is understood to shuttle between endosomal compartments and the plasma membrane, loss of membrane staining would reduce the efflux of free cholesterol to ApoA1 (Phillips 2014). As such, the upper permanent regions of the HF and the telogen HF may have a reduced need for cholesterol efflux when compared to the lower cycling portions.

Indeed, this is supported by the observation that the expression of ABCG1 and SCARB1 are reduced as the HF moves through catagen and into telogen. Whilst the expression of HMGCR remains high, this does not necessarily reflect high levels of cholesterol synthesis activity, which would need to be confirmed using biochemical assays (Zhang et al. 2015).

In addition to cholesterol, ABCG1 mediates the transport of oxysterols (Engel et al. 2007) and intermediates of cholesterol biosynthesis (Wang et al. 2008), which are thought to play a role in preventing oxysterol induced apoptosis (Aye et al. 2010; Engel et al. 2007). The accumulation of cholesterol intermediates in the HF results in hair loss and cycling disorders (Evers et al. 2010; Karnik et al. 2009; Zhang et al. 2017) (as discussed further in Palmer et al. (2020)), yet we detect only low levels of ABCG1 in this study. This may indicate some functional redundancy, with other transport proteins contributing to oxysterol efflux in the HF or suggest the presence of relatively low levels of oxysterols.

ABCA5 is a less well studied ABC transporter, yet its impact on HF biology has been uncovered following the identification of mutations that result in a congenital hypertrichosis (DeStefano et al. 2014). Although the specific allocrite remains to be identified, overexpression of this protein is linked to enhanced cholesterol efflux to ApoE (Fu et al. 2015), and knockout of ABCA5 in murine macrophages lowers HDL efflux (Ye et al. 2010). Within the HF, ABCA5 expression is predominantly cytoplasmic, with the exception of the HS cuticle. Murine studies have indicated localisation to lysosomes and late endosomes (Kubo et al. 2005), and cholesterol has been observed to accumulate within the lysosomes of keratinocytes derived from congenital hypertrichosis patients carrying ABCA5 mutations (DeStefano et al. 2014). The cytoplasmic localisation of ABCA5 identified here could suggest an involvement in intracellular cholesterol transport within human HFs, as described in other cell types.

There are three known isoforms of ABCA5, one encoding a full transporter of 187 kDa, and two half transporters of 99 and 105 kDa. The antibody used in this study detects the 187 and 99 kDa isoforms, however, the higher molecular weight bands detected here could indicate the formation of trimers and tetramers. Additional experimental work using knockout or knockdown cell models are needed to confirm the antibody specificity. Although not previously reported for ABCA5, members of the ABCD (Geillon et al. 2017), ABCG (Xu et al. 2004) and ABCA (Denis et al. 2004) subfamilies have been known to form oligomers. Indeed, ABCA5 was hypothesised by Petry et al. (2003) to form a homo or heterodimer, suggesting additional oligomeric forms of ABCA5 could well exist.

Unlike ABC transporters the unique bi-directional transporter SCARB1 facilitates diffusional cholesterol movement (Shen et al. 2018b). Predominantly responsible for the uptake of cholesterol esters from HDL, SCARB1 can also participate in the efflux of free cholesterol to HDL (Shen et al. 2018b). The protein has several additional functions, including bacterial detection (Guo et al. 2014), and transport of carotenoids (Shyam et al. 2017) and vitamins (Reboul et al. 2011, 2006). As pharmacological inhibition of SCARB1 reduced cholesterol efflux in this study, a role in cholesterol transport in the HF seems likely.

Strikingly, when examining filipin staining patterns, we observed cholesterol striations within the basement membrane of suprabulbar and isthmus regions, but not the bulb (Fig. S2). The extracellular matrix separating the epithelial and mesenchymal compartments of the HF is predominantly composed of collagen IV and laminin (Chermnykh et al. 2018). Previous studies in macrophages have shown that both ABCA1 and ABCG1 can deposit cholesterol within the extracellular matrix (Jin et al. 2015, 2016). These cholesterol deposits have been described as both needles and plates ranging from 4 to 50 µm in size (Suhalim et al. 2012). The cholesterol striations identified in the HF basement membrane may represent an interaction of cholesterol with laminin-332 during cholesterol movement from CTS to ORS (Jones et al. 2018, 2019), or deposits of excess cholesterol monohydrate needles, however, additional experiments are required to confirm this.

Regulation of cholesterol efflux by LXR agonism is well established (Wang et al. 2006; Zanotti et al. 2008); here we show that T0901317 primarily increased ABCA1/ABCG1 expression, as previously reported (Jiang et al. 2006, 2010). Cholesterol efflux from ABCG1 to HDL is well documented (Zanotti et al. 2008) and the transcriptional changes observed correlate with this function in the HF. There is some evidence for LXR activation of SCARB1, however, regulation in steroidogenic tissues is through trophic hormones (Komaromy et al. 1996; Rigotti et al. 1996; Shen et al. 2018b). This is yet to be established in the HF, which has its own capacity for steroidogenesis (Slominski et al. 2013).

The differential patterns of cholesterol transporter expression suggest dynamic changes in cholesterol requirements across the hair cycle. Furthermore, preliminary functional data demonstrate a role for LXR activity in modulating cholesterol transport activity. Future studies focusing on the regulation of cholesterol transport during the hair cycle are needed to shed light on the role of cholesterol homeostasis in hair disorders.

## Supplementary Information

Below is the link to the electronic supplementary material.

Supplementary file 1 (TIF 11952 KB)

Supplementary file 2 (TIF 247426 KB)

Supplementary file 3 (TIF 2020 KB)

Supplementary file 4 (DOCX 43 KB)

## References

[CR1] Aasen T, Izpisua Belmonte JC (2010). Isolation and cultivation of human keratinocytes from skin or plucked hair for the generation of induced pluripotent stem cells. Nat Protoc.

[CR2] Aye IL, Waddell BJ, Mark PJ, Keelan JA (2010). Placental ABCA1 and ABCG1 transporters efflux cholesterol and protect trophoblasts from oxysterol induced toxicity. Biochim Biophys Acta.

[CR3] Bodo E, Biro T, Telek A (2005). A hot new twist to hair biology: involvement of vanilloid receptor-1 (VR1/TRPV1) signaling in human hair growth control. Am J Pathol.

[CR4] Braverman N, Lin P, Moebius FF (1999). Mutations in the gene encoding 3 beta-hydroxysteroid-delta 8, delta 7-isomerase cause X-linked dominant Conradi-Hunermann syndrome. Nat Genet.

[CR5] Chermnykh E, Kalabusheva E, Vorotelyak E (2018). Extracellular matrix as a regulator of epidermal stem cell fate. Int J Mol Sci.

[CR6] Denis M, Haidar B, Marcil M, Bouvier M, Krimbou L, Genest J (2004). Characterization of oligomeric human ATP binding cassette transporter A1. Potential implications for determining the structure of nascent high density lipoprotein particles. J Biol Chem.

[CR7] DeStefano GM, Kurban M, Anyane-Yeboa K (2014). Mutations in the cholesterol transporter gene ABCA5 are associated with excessive hair overgrowth. PLoS Genet.

[CR8] Engel T, Kannenberg F, Fobker M (2007). Expression of ATP binding cassette-transporter ABCG1 prevents cell death by transporting cytotoxic 7beta-hydroxycholesterol. FEBS Lett.

[CR9] Evers BM, Farooqi MS, Shelton JM, Richardson JA, Goldstein JL, Brown MS, Liang G (2010). Hair growth defects in Insig-deficient mice caused by cholesterol precursor accumulation and reversed by simvastatin. J Invest Dermatol.

[CR10] Feingold KR (2009). The outer frontier: the importance of lipid metabolism in the skin. J Lipid Res.

[CR11] Fu Y, Hsiao JH, Paxinos G, Halliday GM, Kim WS (2015). ABCA5 regulates amyloid-beta peptide production and is associated with Alzheimer’s disease neuropathology. J Alzheimers Dis.

[CR12] Geillon F, Gondcaille C, Raas Q (2017). Peroxisomal ATP-binding cassette transporters form mainly tetramers. J Biol Chem.

[CR13] Guo L, Zheng Z, Ai J, Huang B, Li XA (2014). Hepatic scavenger receptor BI protects against polymicrobial-induced sepsis through promoting LPS clearance in mice. J Biol Chem.

[CR14] Haslam IS, El-Chami C, Faruqi H, Shahmalak A, O'Neill CA, Paus R (2015). Differential expression and functionality of ATP-binding cassette transporters in the human hair follicle. Br J Dermatol.

[CR15] Haslam IS, Jadkauskaite L, Szabo IL (2017). Oxidative damage control in a human (Mini-) organ: Nrf2 activation protects against oxidative stress-induced hair growth inhibition. J Invest Dermatol.

[CR16] Hayashi R, Yoshida K, Abe R, Niizeki H, Shimomura Y (2017). First Japanese case of congenital generalized hypertrichosis with a copy number variation on chromosome 17q24. J Dermatol Sci.

[CR17] Incardona JP, Eaton S (2000). Cholesterol in signal transduction. Curr Opin Cell Biol.

[CR18] Jiang YJ, Lu B, Kim P, Elias PM, Feingold KR (2006). Regulation of ABCA1 expression in human keratinocytes and murine epidermis. J Lipid Res.

[CR19] Jiang YJ, Lu B, Tarling EJ (2010). Regulation of ABCG1 expression in human keratinocytes and murine epidermis. J Lipid Res.

[CR20] Jiang Y, Jin H, Zeng Y (2019). A novel mutation in MBTPS2 causes ichthyosis follicularis, alopecia, and photophobia syndrome. Mol Genet Genomic Med.

[CR21] Jin X, Freeman SR, Vaisman B (2015). ABCA1 contributes to macrophage deposition of extracellular cholesterol. J Lipid Res.

[CR22] Jin X, Sviridov D, Liu Y, Vaisman B, Addadi L, Remaley AT, Kruth HS (2016). ABCA1 (ATP-Binding Cassette Transporter A1) Mediates ApoA-I (Apolipoprotein A-I) and ApoA-I Mimetic Peptide Mobilization of Extracellular Cholesterol Microdomains Deposited by Macrophages. Arterioscler Thromb Vasc Biol.

[CR23] Jones E, Marsh S, O'Shaughnessy R, Aumailley M, O'Toole EA, Caley MP (2018). 680 A role for the basement membrane in skin lipid trafficking. J Invest Dermatol.

[CR24] Jones EM, Marsh S, O’Shaughnessy RF (2019). 274 junctional epidermolysis bullosa: bottom up control of the skin barrier?. J Invest Dermatol.

[CR25] Karnik P, Tekeste Z, McCormick TS, Gilliam AC, Price VH, Cooper KD, Mirmirani P (2009). Hair follicle stem cell-specific PPARγ deletion causes scarring alopecia. J Invest Dermatol.

[CR26] Kloepper JE, Sugawara K, Al-Nuaimi Y, Gaspar E, van Beek N, Paus R (2010). Methods in hair research: how to objectively distinguish between anagen and catagen in human hair follicle organ culture. Exp Dermatol.

[CR27] Komaromy M, Azhar S, Cooper AD (1996). Chinese hamster ovary cells expressing a cell surface-anchored form of hepatic lipase. Characterization of low density lipoprotein and chylomicron remnant uptake and selective uptake of high density lipoprotein-cholesteryl ester. J Biol Chem.

[CR28] Kubo Y, Sekiya S, Ohigashi M (2005). ABCA5 resides in lysosomes, and ABCA5 knockout mice develop lysosomal disease-like symptoms. Mol Cell Biol.

[CR29] Langan EA, Philpott MP, Kloepper JE, Paus R (2015). Human hair follicle organ culture: theory, application and perspectives. Exp Dermatol.

[CR30] Lee J, Tumbar T (2012). Hairy tale of signaling in hair follicle development and cycling. Semin Cell Dev Biol.

[CR31] Limat A, Noser FK (1986). Serial cultivation of single keratinocytes from the outer root sheath of human scalp hair follicles. J Invest Dermatol.

[CR32] Maxfield FR, Wüstner D (2012) Analysis of cholesterol trafficking with fluorescent probes. In. Elsevier, pp 367–393. doi:10.1016/b978-0-12-386487-1.00017-1 10.1016/B978-0-12-386487-1.00017-1 PMC3626500 22325611

[CR33] Nakaya K, Tohyama J, Naik SU, Tanigawa H, MacPhee C, Billheimer JT, Rader DJ (2011). Peroxisome proliferator-activated receptor-alpha activation promotes macrophage reverse cholesterol transport through a liver X receptor-dependent pathway. Arterioscler Thromb Vasc Biol.

[CR34] Neufeld EB, Remaley AT, Demosky SJ (2001). Cellular localization and trafficking of the human ABCA1 transporter. J Biol Chem.

[CR35] Nieland TJF, Chroni A, Fitzgerald ML, Maliga Z, Zannis VI, Kirchhausen T, Krieger M (2004). Cross-inhibition of SR-BI- and ABCA1-mediated cholesterol transport by the small molecules BLT-4 and glyburide. J Lipid Res.

[CR36] Oh JW, Kloepper J, Langan EA (2016). A guide to studying human hair follicle cycling in vivo. J Invest Dermatol.

[CR37] Palmer MA, Blakeborough L, Harries M, Haslam IS (2020). Cholesterol homeostasis: Links to hair follicle biology and hair disorders. Exp Dermatol.

[CR38] Payne AH, Hales DB (2004). Overview of steroidogenic enzymes in the pathway from cholesterol to active steroid hormones. Endocr Rev.

[CR39] Petry F, Kotthaus A, Hirsch-Ernst KI (2003). Cloning of human and rat ABCA5/Abca5 and detection of a human splice variant. Biochem Biophys Res Commun.

[CR40] Phillips MC (2014). Molecular mechanisms of cellular cholesterol efflux. J Biol Chem.

[CR41] Philpott MP (2018). Culture of the human pilosebaceous unit, hair follicle and sebaceous gland. Exp Dermatol.

[CR42] Philpott M, Green MR, Kealey T (1989). Studies on the biochemistry and morphology of freshly isolated and maintained rat hair follicles. J Cell Sci.

[CR43] Reboul E, Klein A, Bietrix F (2006). Scavenger receptor class B type I (SR-BI) is involved in vitamin E transport across the enterocyte. J Biol Chem.

[CR44] Reboul E, Goncalves A, Comera C (2011). Vitamin D intestinal absorption is not a simple passive diffusion: evidences for involvement of cholesterol transporters. Mol Nutr Food Res.

[CR45] Rigotti A, Edelman ER, Seifert P (1996). Regulation by adrenocorticotropic hormone of the in vivo expression of scavenger receptor class B type I (SR-BI), a high density lipoprotein receptor, in steroidogenic cells of the murine adrenal gland. J Biol Chem.

[CR46] Romano MT, Tafazzoli A, Mattern M (2018). Bi-allelic Mutations in LSS, Encoding Lanosterol Synthase, Cause Autosomal-Recessive Hypotrichosis Simplex. Am J Hum Genet.

[CR47] Russell LE, Harrison WJ, Bahta AW, Zouboulis CC, Burrin JM, Philpott MP (2007). Characterization of liver X receptor expression and function in human skin and the pilosebaceous unit. Exp Dermatol.

[CR48] Sankaranarayanan S, Kellner-Weibel G, de la Llera-Moya M, Phillips MC, Asztalos BF, Bittman R, Rothblat GH (2011). A sensitive assay for ABCA1-mediated cholesterol efflux using BODIPY-cholesterol. J Lipid Res.

[CR49] Shen W-J, Asthana S, Kraemer FB, Azhar S (2018). Scavenger receptor B type 1: expression, molecular regulation, and cholesterol transport function. J Lipid Res.

[CR50] Shen WJ, Azhar S, Kraemer FB (2018). SR-B1: A Unique Multifunctional receptor for cholesterol influx and efflux. Annu Rev Physiol.

[CR51] Shyam R, Vachali P, Gorusupudi A, Nelson K, Bernstein PS (2017). All three human scavenger receptor class B proteins can bind and transport all three macular xanthophyll carotenoids. Arch Biochem Biophys.

[CR52] Slominski A, Zbytek B, Nikolakis G (2013). Steroidogenesis in the skin: implications for local immune functions. J Steroid Biochem Mol Biol.

[CR53] Sticozzi C, Belmonte G, Pecorelli A (2012). Cigarette smoke affects keratinocytes SRB1 expression and localization via H2O2 production and HNE protein adducts formation. PLoS ONE.

[CR54] Suhalim JL, Chung CY, Lilledahl MB, Lim RS, Levi M, Tromberg BJ, Potma EO (2012). Characterization of cholesterol crystals in atherosclerotic plaques using stimulated Raman scattering and second-harmonic generation microscopy. Biophys J.

[CR55] Thiboutot D, Jabara S, McAllister JM, Sivarajah A, Gilliland K, Cong Z, Clawson G (2003). Human skin is a steroidogenic tissue: steroidogenic enzymes and cofactors are expressed in epidermis, normal sebocytes, and an immortalized sebocyte cell line (SEB-1). J Invest Dermatol.

[CR56] Wang N, Ranalletta M, Matsuura F, Peng F, Tall AR (2006). LXR-induced redistribution of ABCG1 to plasma membrane in macrophages enhances cholesterol mass efflux to HDL. Arterioscler Thromb Vasc Biol.

[CR57] Wang N, Yvan-Charvet L, Lutjohann D, Mulder M, Vanmierlo T, Kim TW, Tall AR (2008). ATP-binding cassette transporters G1 and G4 mediate cholesterol and desmosterol efflux to HDL and regulate sterol accumulation in the brain. FASEB J.

[CR58] Wertz PW (2000). Lipids and barrier function of the skin. Acta Derm Venereol Suppl (Stockh).

[CR59] Xu J, Liu Y, Yang Y, Bates S, Zhang JT (2004). Characterization of oligomeric human half-ABC transporter ATP-binding cassette G2. J Biol Chem.

[CR60] Ye D, Meurs I, Ohigashi M (2010). Macrophage ABCA5 deficiency influences cellular cholesterol efflux and increases susceptibility to atherosclerosis in female LDLr knockout mice. Biochem Biophys Res Commun.

[CR61] Zanotti I, Poti F, Pedrelli M (2008). The LXR agonist T0901317 promotes the reverse cholesterol transport from macrophages by increasing plasma efflux potential. J Lipid Res.

[CR62] Zhang X, Song Y, Feng M (2015). Thyroid-stimulating hormone decreases HMG-CoA reductase phosphorylation via AMP-activated protein kinase in the liver. J Lipid Res.

[CR63] Zhang J, Wang Y, Cheng R, Ni C, Liang J, Li M, Yao Z (2016). Novel MBTPS2 missense mutation causes a keratosis follicularis spinulosa decalvans phenotype: mutation update and review of the literature. Clin Exp Dermatol.

[CR64] Zhang D, Tomisato W, Su L (2017). Skin-specific regulation of SREBP processing and lipid biosynthesis by glycerol kinase 5. Proc Natl Acad Sci U S A.

[CR65] Zhao C, Dahlman-Wright K (2010). Liver X receptor in cholesterol metabolism. J Endocrinol.

