# Supplementary figures and images for: Localisation and regulation of cholesterol transporters in the human hair follicle: mapping changes across the hair cycle
Source: Histochem Cell Biol. 2021 Jan 6;155(5):529–45. doi: 10.1007/s00418-020-01957-8 (PMC8134313; doi:10.1007/s00418-020-01957-8)

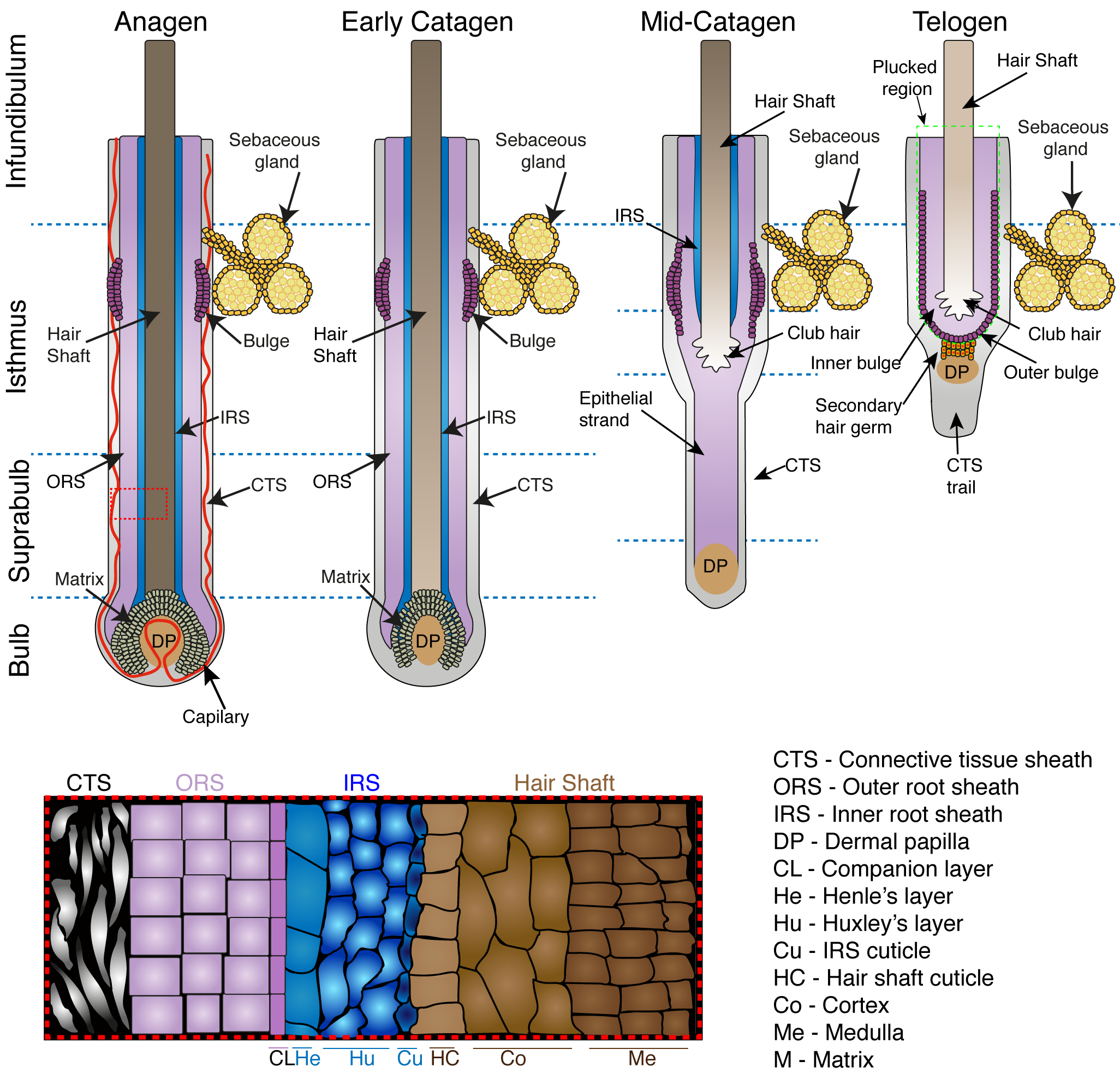

Supplement: Supplementary file 1 — (TIF 11952 KB) [file 418_2020_1957_MOESM1_ESM.tif]

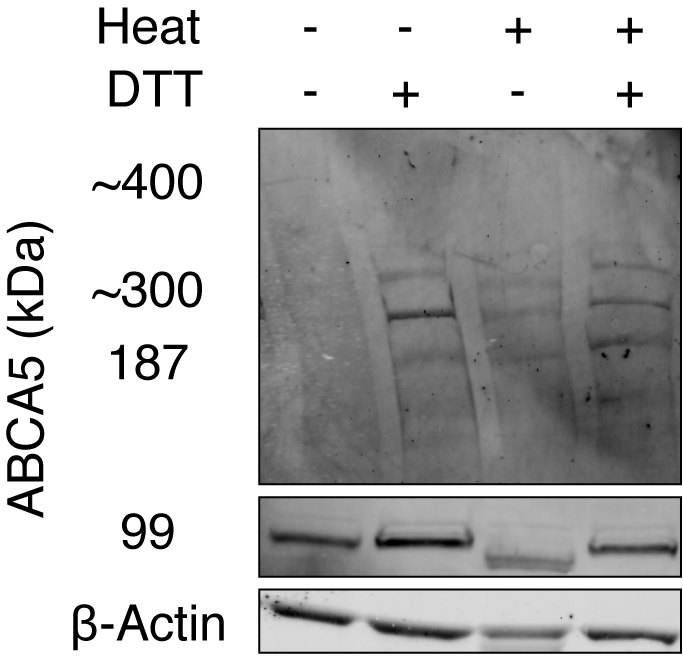

Supplement: Supplementary file 3 — (TIF 2020 KB) [file 418_2020_1957_MOESM3_ESM.tif]
