# Supplementary material for: Localisation and regulation of cholesterol transporters in the human hair follicle: mapping changes across the hair cycle
Source: Histochem Cell Biol. 2021 Jan 6;155(5):529–45. doi: 10.1007/s00418-020-01957-8 (PMC8134313; doi:10.1007/s00418-020-01957-8)
Supplement: Supplementary file 4 — (DOCX 43 KB) [file 418_2020_1957_MOESM4_ESM.docx]

**Supplementary material**

**Localisation and regulation of cholesterol transporters in the human hair follicle: mapping changes across the hair cycle.**

Megan A. Palmer^1*^, Eleanor Smart^2^ and Iain S. Haslam^1^

^1^Department of Biological Sciences, School of Applied Sciences, University of Huddersfield, Huddersfield, HD1 3DH, UK

^2^ Centre for Dermatology Research, School of Biological Sciences, University of Manchester & NIHR Biomedical Research Centre, Manchester, M13 9PT, UK

* corresponding author – megan.palmer@hud.ac.uk

**Table S1: Details of hair follicle donors**

|  | **Donor** | **Anagen**  [Age (ID)] | **Early Catagen**  [Age (ID)] | **Mid-Catagen**  [Age (ID)] | **Telogen**  [Age (ID)] |
| --- | --- | --- | --- | --- | --- |
| **ABCA1** | 1  2  3  4 | 65 (002)  69 (019)  **59 (022)**  - | **65 (002)**  25 (003)  55 (020)  - | 60 (018)  **69** **(019)**  -  - | **(007)***  (009) *  (013) *  30 (015) |
| **ABCG1** | 1  2  3  4 | 54 (001)  69 (019)  **59 (022)**  - | 25 (003)  60 (004)  **69 (019)**  - | 60 (018)  **69** **(019)**  -  - | **(013) ***  (014) *  30 (015)  - |
| **ABCA5** | 1  2  3  4 | **65 (002)**  69 (019)  59 (022)  - | **25 (003)**  60 (004)  69 (019)  - | 60 (018)  **69** **(019)**  -  - | **(005) ***  (009) *  30 (015)  - |
| **SCARB1** | 1  2  3  4 | 65 (002)  **60 (018)**  69 (019)  - | **65 (002)**  60 (004)  69 (019)  - | 60 (018)  **69** **(019)**  -  - | (007) *  (013) *  **(014) ***  - |
| **HMGCR** | 1  2  3  4 | 65 (002)  69 (019)  **59 (022)**  - | 25 (003)  **55 (006)**  60 (018)  - | 60 (018)  **69** **(019)**  -  - | **(009) ***  (012) *  30 (015)  - |
| **Filipin** | 1  2  3  4 | **65 (002)**  69 (019)  59 (022)  - | **25 (003)**  60 (004)  60 (018)  - | 60 (018)  **69** **(019)**  -  - | (005) *  (009) *  **30 (015)**  - |

Bold text indicates the donor from which images in the manuscript are shown, * donor age not supplied. ID is the donor number.

**Table S2: Summary of immunofluorescence protocols**

| **Antibody** | **Fixative** | **Block** | **Wash** | **Primary dilution** | **Secondary Antibody** | **Secondary dilution** | **Primary product code** |
| --- | --- | --- | --- | --- | --- | --- | --- |
| ABCA1 | 1:1 Acetone:  Methanol | 2.5% NHS | TBS-T | 1:25 | VectaFluor excel anti-Mouse 488 | N/A | ab18180 (abcam) |
| ABCA5 | 100% Acetone | 10% NGS | PBS | 1:200 | Goat anti-Rabbit 594 | 1:200 | ab99953 (abcam) |
| ABCG1 | 1:1 Acetone:  Methanol | 2.5% NHS | TBS-T | 1:50 | VectaFluor excel anti-Rabbit 488 | N/A | ab52617 (abcam) |
| SCARB1 | 100% Acetone | 10% NGS | PBS | 1:200 | Goat anti-Rabbit 594 | 1:1000 | ab217318 (abcam) |
| HMGCR | 100% Acetone | 10% NGS | PBS | 1:100 | Goat anti-Rabbit 594 | 1:200 | 13533-1-AP  (proteintech®) |
| Laminin-332 | Any of the above or  4% paraformaldehyde PFA) | 10% NGS | PBS | 1:1000 | Goat anti-Mouse 488 or 568 | 1:200 | ab78286 (abcam) |
| CD200 |  | 10% NGS | PBS/TBS | 1:200 | Goat anti-Mouse 488 | 1:200 | MCA1960GA (Bio-Rad, California, USA) |
| Filipin |  | 10 mg/ml glycine | PBS | 100 µg/ml | N/A | N/A | F4767  (Sigma) |

**The hair follicle**

The HF is a cyclical mini-organ undergoing periods of growth (anagen), regression (catagen), and relative quiescence (telogen) (Geyfman et al. 2015; Oh et al. 2016; Schneider et al. 2009). Comprised of distinct cell layers (Fig. S1, red box) of epithelial derived keratinocytes with differential expression of specific keratins and morphologies (Langbein and Schweizer 2005), surrounded by a mesenchymal layer of fibroblasts (connective tissue sheath) and the dermal papilla (DP) located within the bulb of the HF.

**Fig. S1: Schematic representation of the hair cycle and anatomy**. The HF is comprised of concentric layers of keratinocytes encompassing of the hair shaft, inner root sheath (IRS) and outer root sheath (ORS). Surrounded by a fibroblast layer the connective tissue sheath (CTS), containing the vasculature supply to the HF. The bulb region of the HF is located proximally and contains the signalling centre of the HF, dermal papilla (DP), and highly proliferative matrix keratinocytes. Distal to the bulb the HF can be broken down into the suprabulb, isthmus and infundibulum regions. Within the isthmus the ORS contains the K15/CD200+ bulge region of keratinocytes, proximal to the sebaceous gland. As a cyclical mini-organ that goes through periods of growth (anagen), apoptosis mediated regression (catagen) and relative quiescence (telogen). Early catagen is represented by a loss of pigmentation in the hair shaft, loss of matrix keratinocytes and a transition from the onion shaped DP in anagen to an almond for early catagen. Further regression occurs during mid-catagen to reveal the epithelial stand and formation of the club hair, along with migration of bulge keratinocytes. During telogen the DP is condensed, bulge keratinocytes have migrated and surrounded the club hair (outer bulge) along with the production of the secondary hair germ and a CTS trail present. Plucked telogen follicles are represented by the dashed green line, in which the fibroblast layers, sebaceous gland and secondary hair germ are retained in the scalp. The red dashed box in anagen represents the alterations in cell morphology of the individual HF layers, including the companion layer (CL), three layers of the IRS (Henle’s; He, Huxley’s; Hu, Cuticle; Cu) and hair shaft (hair shaft cuticle; HC, Cortex; Co, Medulla; Me).

**Fig. S2: Cholesterol striations are unique to the basement membrane of hair follicles.** Freshly isolated anagen, mid-catagen or epidermis. Co-localisation of Filipin (grey) with Laminin-332 (red). Epidermis shows cholesterol staining within the membranes of the epidermis, whereas epidermal basement membrane as shown by laminin-332 staining does not contain striations of cholesterol. Striations of cholesterol within the basement membrane of anagen (red arrows) and mid-catagen (green arrows). Scale bar 50 µm, red dashed box delineates magnified area, scale bars 5 µm, green dashed box delineates magnified area from mid-catagen. *Anagen is representative field of view for magnified image of upper suprabulbar area and not actual image.

**Fig. S3:** Oligomeric ABCA5 of 400 kDa is present in non-reducing conditions. Western blot for ABCA5 with the addition of higher concentration of reducing agent 500 µM and heating. Both higher concentration of DTT and heat results in the removal of 400 kDa ABCA5. 187 kDa ABCA5 is not present during non-reducing conditions.

**References**

Geyfman M, Plikus MV, Treffeisen E, Andersen B, Paus R (2015) Resting no more: re-defining telogen, the maintenance stage of the hair growth cycle. Biol Rev Camb Philos Soc 90:1179-1196. doi:10.1111/brv.12151

Langbein L, Schweizer J (2005) Keratins of the human hair follicle. Int Rev Cytol 243:1-78. doi:10.1016/S0074-7696(05)43001-6

Oh JW, Kloepper J, Langan EA et al. (2016) A Guide to Studying Human Hair Follicle Cycling In Vivo. J Invest Dermatol 136:34-44. doi:10.1038/JID.2015.354

Schneider MR, Schmidt-Ullrich R, Paus R (2009) The hair follicle as a dynamic miniorgan. Curr Biol 19:R132-142. doi:10.1016/j.cub.2008.12.005
